# Efficient Extraction and Analysis of Wheat Straw Lignin by Response Surface Methodology

**DOI:** 10.3390/polym16202935

**Published:** 2024-10-19

**Authors:** Yongke Wang, Xiao-Feng Sun, Jiayi Chen, Sihai Hu, Ran Sun

**Affiliations:** 1 School of Chemistry and Chemical Engineering, Northwestern Polytechnical University, Xi’an 710072, China; shidaijiuji@mail.nwpu.edu.cn (Y.W.); chenjiayi@mail.nwpu.edu.cn (J.C.); sunran@nwpu.edu.cn (R.S.); 2 Research & Development Institute of Northwestern Polytechnical University in Shenzhen, Shenzhen 518063, China

**Keywords:** lignin extraction, formic acid/acetic acid solvent system, response surface methodology, extraction optimization

## Abstract

To enhance the high-value utilization of straw waste and achieve efficient lignin extraction, wheat straw was selected as the feedstock for investigating the effects of reaction temperature, reaction time, solid–liquid ratio, and formic acid concentration on lignin yield using a formic acid/acetic acid solvent system. A single-factor experimental design was initially employed, followed by optimization using the response surface methodology. Additionally, a kinetic model was developed to describe lignin extraction kinetics in the formic acid/acetic acid system. The structural characteristics and thermal stability of the extracted lignin were analyzed via FTIR, UV spectroscopy, and TGA. The findings indicate that increasing reaction temperature, reaction time, solid–liquid ratio, and formic acid content all significantly enhanced lignin extraction yield from wheat straw, with the primary influencing factors being reaction temperature > solid–liquid ratio > reaction time > formic acid content. The optimal extraction conditions were identified at a reaction temperature of 90 °C, a reaction time of 3.5 h, a solid–liquid ratio of 1:16.5, and a formic acid content of 86.2 wt.%, yielding a lignin content of 79.83%. The analytical results demonstrated that the extracted lignin preserved the structural integrity of the original lignin and exhibited good thermal stability.

## 1. Introduction

As a natural high-molecular-weight polymer, lignin is extensively distributed within plant cell walls and represents a biomass resource of significant abundance. In China, the annual production of straw by-products is approximately 700 million tons, with lignin comprising about 140 million tons of this total. However, the prevailing methods of lignin treatment predominantly involve incineration or direct discharge as “black liquor” in the paper industry, leading to substantial resource wastage and environmental pollution due to its underutilization [1,2].

The techniques for lignin separation have been under development since the 1930s, with a history spanning over 190 years [3]. Based on the underlying separation principles, lignin extraction methods can be broadly classified into two categories: the first approach dissolves hemicellulose and collects cellulose within the plant, thereby isolating lignin as an insoluble component via filtration [4]; the second approach involves the dissolution of lignin as a soluble component, followed by filtration to remove other insoluble components, thus achieving lignin separation [5]. Lignin is abundantly present within the various tissues of straw. By extracting lignin from these sources and subjecting it to processes such as pyrolysis, oxidation, or other chemical transformations, it can be decomposed into aromatic compounds or prepared to nanolignins [6,7]. These compounds can then be utilized to produce bio-based plastics, resins, and composite materials [8]. These materials are not only renewable but also biodegradable, offering a sustainable alternative to traditional petroleum-based plastics and materials. This approach aligns with the goals of green energy development and contributes to advancing sustainable practices. In light of the global depletion of fossil resources, the efficient utilization of biomass resources, particularly the integrated use of plant-derived components such as hemicellulose and lignin, has become increasingly crucial. Traditional lignin separation techniques, such as the alkali and acid methods, though widely employed in industrial applications, exhibit several drawbacks, including low resource utilization efficiency, high equipment demands, and significant environmental impact [9,10,11].

In contrast, the organic acid method has emerged as a promising lignin separation technique, garnering attention due to its ability to produce lignin with a structure closely resembling natural lignin, alongside a lower molecular weight and favorable industrial application potential [12]. This method offers several advantages, including relatively mild reaction conditions, effective lignin dissolution by organic acids, and the potential for acid recovery and reuse, thereby mitigating costs and reducing environmental impact [13]. To achieve the high-value utilization of straw waste and to facilitate lignin extraction, this study employed a formic acid/acetic acid system for the efficient extraction of wheat straw lignin by response surface methodology. This process can cleave the α-aryl ether and β-aryl ether bonds within the lignin structure effectively, enabling the detachment and dissolution of lignin from straw in the formic acid/acetic acid system [14]. The factors influencing the lignin extraction rate within the formic acid/acetic acid system were systematically investigated, and the data were optimized using response surface methodology to establish the optimal extraction conditions [15]. Finally, the structural characteristics and stability of the extracted lignin were analyzed using Fourier Transform Infrared Spectroscopy (FTIR), Ultraviolet-Visible Spectroscopy (UV-Vis), and Thermogravimetric-Differential Thermogravimetric Analysis (TGA), providing a theoretical foundation and technical support for the lignin.

## 2. Materials and Methods

### 2.1. Raw Material and Chemicals

Wheat straw was collected from local crops (Xi’an, China) and was naturally air-dried. The dried straw was pulverized into a 20–40 mesh particle sample by a plant mill, and the sample was washed with purified water and dried in an oven at 60 °C for 36 h; then, the dried sample was placed in a sealed bag. Formic acid and ethylene diamine tetraacetic acid (EDTA) were purchased from Tianjin Jinbei Fine Chemical Co., Ltd. (Tianjin, China), and acetic acid, Na_2_HPO_4_, and Na_2_B_4_O_7_·2H_2_O were purchased from Tianjin Komi Chemical Reagent Factory (Tianjin, China). All chemicals are analytical grade.

### 2.2. Analysis of Wheat Straw Composition

The contents of cellulose, hemicellulose, lignin, and ash in wheat straw were analyzed using Van Soest method [16]. In the first step, the pulverized wheat straw granules were put into an Erlenmeyer flask (with a condensing device), and then 0.3 wt.% EDTA solution was added and reacted at 90 °C for 1 h. After the solution was cooled, the sample was filtered with gauze and washed, and the cleaned filter cake was dried in an oven at 60 °C.

In the second step, the EDTA-treated wheat straw was subjected to Soxhlet extraction according to GB/T2677.6 [17]. A certain amount of treated straw sample was placed in a gauze bag, which was tied with a thread and put into the Soxhlet extractor. The composition of the phenyl alcohol mixture was benzene:ethanol (2:1, *v*/*v*); after being extracted at 85 °C for 24 h, the sample was air-dried and then placed in an oven to dry.

The third step is to prepare the neutral detergent solution. A total of 2.28 g of anhydrous sodium hydrogen phosphate (Na_2_HPO_4_) was dissolved in distilled water by heating and was recorded as A solution. A total of 9.3 g of EDTA and 3.4 g of sodium borate (Na_2_B_4_O_7_·2H_2_O) were dissolved in distilled water, then added to 15 g of sodium lauryl sulfate and 5 mL of ethylene glycol diether and recorded as B solution. After mixing the A solution and the B solution, the solution was adjusted to 1000 mL in a volumetric flask to obtain a neutral detergent with a pH value of 7.

In the fourth step, 1 g sample was put into 200 mL iodine measuring bottle, added 100 mL neutral detergent, then placed in boiling water bath, heated at 100 °C for 1 h, then taken out and filtered with sand core funnel. The residue obtained by filtration was washed with deionized water and acetone to obtain residue 1. Residue 1 was placed in a vacuum drying oven, dried to a constant weight at 60 °C, and weighed to W_1_.

In the fifth step, residue 1 was placed in a 200 mL iodine measuring flask, and 100 mL of the prepared 2M hydrochloric acid solution was added, stirred, and uniformly mixed, placed in a boiling water bath, heated in a constant-temperature water bath at 100 °C for 50 min, and then filtered through a sand core funnel. The filter residue was washed with water and acetone until the pH value was neutral, and residue 2 was obtained. The washed residue was placed in a dry box, dried to a constant weight at 60 °C, weighed, and recorded as W_2_.

In the sixth step, residue 2 was placed in a 200 mL iodine measuring flask, 10 mL of the prepared 72% sulfuric acid solution was added, and the hydrolysis was carried out at 20 °C for 3 h. Then, 90 mL of deionized water was added, keeping it at room temperature overnight, and the core funnel was used for the next day. After filtration and washing the residue with deionized water to pH 6.5 to obtain residue 3, residue 3 was placed in a vacuum drying oven, and the temperature was set at 60 °C. It was dried to constant weight and then weighed and recorded as W_3_.

In the seventh step, residue 3 was heated at 550 °C to obtain ash, and the ash weight was recorded as W_4_. The calculation formulas are as follows:

(1) $$Hemicellulose(\%)=(w_{1}-w_{2})/1\;g\times 100\%$$

(2) $$Cellulose(\%)=(w_{2}-w_{3})/1\;g\times 100\%$$

(3) $$Lignin(\%)=(w_{3}-w_{4})/1\;g\times 100\%$$

### 2.3. Lignin Extraction Experiment

Optimal experimental conditions for lignin extraction were determined by the single-factor analysis. A total of 3 g of wheat straw was added into a 100 mL round-bottom flask with the configured formic acid reaction solvent according to a certain solid–liquid ratio and reacted for a certain time at a certain temperature. After the reaction was completed, the reaction slurry was filtered, the residue was washed three times with a corresponding ratio of formic acid/acetic acid solvent, the filtrate was collected and concentrated under reduced pressure at 50 °C, and then 150 mL of deionized water was added to precipitate. After separation by a centrifuge (5000 r/min, 10 min), lignin was obtained as insoluble material and washed three times with deionized water, and the obtained lignin was dried under vacuum at 60 °C.

### 2.4. Experiment Design and Optimization

Based on the results of single-factor experiments, the four influencing factors of reaction temperature, reaction time, solid–liquid ratio, and formic acid/acetic acid ratio were selected to be optimized using the response surface method. Through the previous investigation of the factors affecting the yield of straw lignin, the experimental factors were determined, as shown in Table 1.

Using the four-factor five-central composite design (CCD) optimization design method in response surface, the lignin extraction yields (%LEY) were chosen as the response value (expressed by Y), and the Design-Expert 8.0.5b software was applied. The number of experimental groups was 27 groups, as shown in Table 2.

For the experimental data in Table 2, a quadratic polynomial 3-1 model (4) was used for fitting the following:

(4) $$\begin{array}{c} Y_{i}=\beta_{0}+\beta_{1}X_{1}+\beta_{2}X_{2}+\beta_{3}X_{3}+\beta_{4}X_{4}+\beta_{11}X_{1}^{2}+\beta_{22}X_{2}^{2}+\beta_{33}X_{3}^{2}+\beta_{44}X_{4}^{2}+\beta_{12}X_{1}X_{2}+\beta_{13}X_{1}X_{3}\\ +\beta_{14}X_{1}X_{4}+\beta_{23}X_{2}X_{3}+\beta_{24}X_{2}X_{4}+\beta_{34}X_{3}X_{4} \end{array}$$

In Formula (4), Y_i_ is the lignin yield response value, X_i_ is the experimental influencing factor, β_0_, β_1_, β_2_, β_3_, and β_4_ are the first term coefficients, β_11_, β_22_, β_33_, and β_44_ are the quadratic coefficients, and β_12_, β_13_, β_14_, β_23_, β_24_, and β_34_ are interaction term coefficients.

### 2.5. Characterization of Extracted Lignin

To characterize the change of chemical structure of the extracted lignin, FT-IR, TGA, and UV-Vis analysis were conducted in this study. The molecular weight of the extracted lignin using a formic acid/acetic acid system had been studied by our group and other scientists [18], and the formic acid/acetic acid system had no substantial influences on the molecular weight of the lignin from wheat straw.

The lignin sample was ground into a powder and dried at 60 °C for 12 h. An appropriate amount of the sample to be tested and a small amount of KBr powder were ground in an agate mortar, the ground mixed sample was pressed into a transparent sheet by a tableting machine, and then infrared analysis was performed by a Nicolet 510 spectrophotometer (Thermo Scientific, Waltham, MA, USA). The infrared analysis scan range is 400–5000 cm^−1^, the number of scans is 64 times, and the resolution is 4 cm^−1^.

Thermogravimetric analysis was performed on a Mettler-TGA/SDTA thermogravimetric analyzer. The 10–20 mg sample was weighted into the crucible, selecting helium as the carrier gas, the carrier gas flow rate was 10 mL/min, and the heating rate was 10 °C/min. The test temperature range was 30–950 °C.

A sample of 10 mg of lignin was dissolved in dimethyl sulfoxide with dimethyl sulfoxide as a blank, and the UV-Vis spectrum of the extracted lignin was measured in the range of 200–700 nm by the UV-Vis spectrophotometer (Shanghai Jingke 752N, Shanghai, China).

### 2.6. Statistical Analysis

All data were collected from over three independent experiments, and differences between groups were analyzed by two-way ANOVA Tukey’s test using GraphPad Prism 9.5 (GraphPad Software Inc., San Diego, CA, USA).

## 3. Results and Discussion

### 3.1. Raw Material Composition Analysis

The analysis result of the wheat straw composition is shown in Table 3, containing 48.34% cellulose, 18.86% lignin, 22.64% hemicellulose, and 2.56% ash. The deviations of these contents from their respective means were all less than 7.8%.

### 3.2. Effects of Four Experimental Influencing Factors

**Figure 1:**
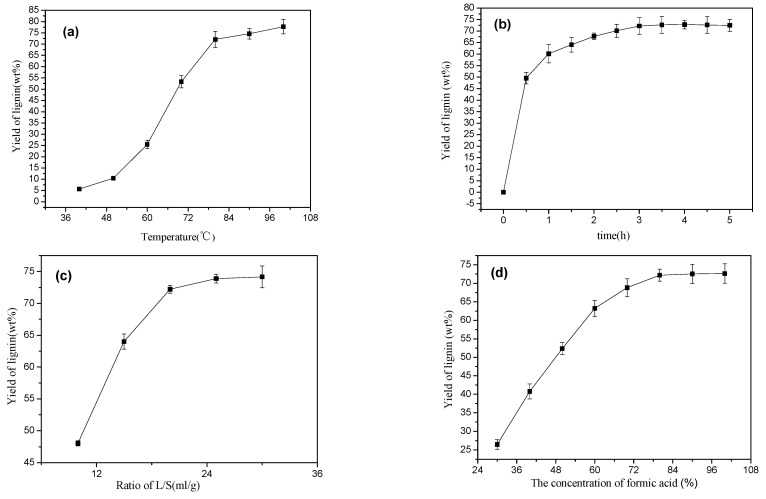
Effects of four experimental influencing factors: (**a**) reaction temperature, (**b**) reaction time, (**c**) liquid-solid ratio, (**d**) formic acid concentration.

When the reaction time was 3 h, the solid–liquid ratio was 1:20, and the formic acid concentration was 80 wt.%, the effect of reaction temperatures on the yield of lignin was studied. The experimental results are shown in Figure 1a.

It can be seen from Figure 1a that the yield of lignin increased sharply with an increase in the reaction temperature before the reaction temperature reached 80 °C. When the temperature reached 80 °C, the yield of lignin rose to 72.09%. Because the ether bond or ester bond between lignin and hemicellulose was broken, the lignin was dissolved [19]. After the reaction temperature was raised to 80 °C, the increase in lignin yield tended to be gentle, which may be due to the solvent–solute system reaching a plateau, preventing further progress under those conditions, and a side reaction such as condensation and rearrangement of lignin may have taken place, affecting the progress of the delignification reaction [20]. This indicated that the temperature of 80 °C was suitable for the lignin extraction experiment.

When the reaction temperature was 80 °C, the solid–liquid ratio was 1:20, and the formic acid concentration was 80 wt.%, the effect of reaction time on the yield of lignin was studied. The experimental results are shown in Figure 1b.

It can be seen from Figure 1b that the extraction process of lignin can be divided into two stages: the first stage was a rapid delignification reaction stage of 0–0.5 h, and the yield of lignin rapidly reached 55.63%. It is mainly comprised of the hydrolysis, cracking, and removal stages of amorphous lignin that is not tightly bound to carbohydrates, such as cellulose and hemicellulose in straw. The extracted lignin was mainly low-molecular-weight components, which are easy to react and easy to dissolve [21]; the second stage was a slow lignin extraction stage of 0.5–3 h. The increase of lignin yield in this stage was much lower than the first stage, and only 16.58% lignin was obtained because the delignification at this stage was mainly macromolecular lignin with relatively large molecular weight and low reactivity, and its structure was relatively stable and difficult to react [22]. Moreover, some of the lignin that was dissolved was reabsorbed into the cellulose, and a part of the lignin could form a new chemical bond with hemicellulose, such as a hydrogen bond, which increased the difficulty of the delignification [23]. When the reaction time reached 3 h, some lignin remained in the crude fiber, the molecular structure of which was more stable and more difficult to extract, and this may be related to the rearrangement or condensation reaction of lignin [24]. Therefore, it is appropriate to consider the reaction time for 3 h.

When the reaction temperature was 80 °C, the reaction time was 3 h, and the formic acid concentration in the formic acid/acetic acid system was 80 wt.%, the effect of the solid–liquid ratio on the yield of lignin was studied. The experimental results are shown in Figure 1c.

When the liquid–solid ratio was less than 20:1, the yield of lignin rapidly increased to 72.23%. This was because less solvent liquid was used, the interaction between the solvent and the straw was not sufficient, the straw was subjected to thermal expansion and solvent evaporation, and an increase in the amount of the solvent can dilute the concentration of lignin in the solution and increase the amount of dissolved lignin [25]. At the same time, an increase in the solvent amount can increase the contact between the organic acid molecules and the straw. When the solid–liquid ratio reached 20:1, the increase in the yield of lignin was relatively slow. This was because a large amount of lignin was dissolved, there was less remaining lignin in the straw, and the remaining lignin and cellulose may form an LCC structure that is not easy to remove [26]. Therefore, the best liquid–solid ratio of 20:1 was appropriate.

When the reaction temperature was 80 °C, the reaction time was 3 h, and the liquid–solid ratio was 20:1, the effect of formic acid content on the yield of lignin was studied. The experimental results are shown in Figure 1d.

As the formic acid content of the organic acid system increased, the yield of lignin also rose. When the organic acid was used to treat the straw, due to the catalytic action of H^+^ in the solvent, the intra- and inter-molecular cleavage mode of the lignin was mainly the cleavages of the α-aryl ether bond and the β-aryl ether bond [27]. Before the content of formic acid reached 80 wt.%, with the increase of formic acid content, the acidity in the formic acid/acetic acid system became stronger, the catalytic effect of H^+^ was stronger, and the α-aryl ether bond and β-aryl ether bond of the lignin were broken. The stronger the fracture, the more intense the hydrolysis and degradation reaction between the lignin macromolecule and the hemicellulose and other carbon hydrates, and the yield of lignin increased [28]. When the formic acid content increased to 80 wt.%, the yield of lignin reached 72.4%. Due to the limited acidity of formic acid and acetic acid, the acid strength of the solvent changed slowly even if the proportion of formic acid was further increased. The lignin was mostly dissolved, and the remaining residual lignin was difficult to remove, resulting in the extraction rate of lignin tending to be balanced. Therefore, the best ratio of formic acid:acetic acid was 4:1.

### 3.3. Response Surface Analysis

The Design-Expert software was applied to the multiple regression fitting of the experimental data in Table 2, and the quadratic polynomial model with the response value of lignin yield was obtained [29]. The model was as follows:

(5) $$\begin{array}{c} Y=72.2+1135X_{1}+3.58X_{2}+5.28X_{3}+2.22X_{4}-3.59X_{1}^{2}-1.22X_{2}^{2}-2.90X_{3}^{2}-1.16X_{4}^{2}-3.42X_{1}X_{2}\\ -3.22X_{1}X_{3}-0.39X_{1}X_{4}+0.71X_{2}X_{3}-0.15X_{2}X_{4}+0.26X_{3}X_{4} \end{array}$$

The mathematical model of formic acid/acetic acid extraction was analyzed by ANOVA to test the validity of the equation and the regression coefficients of each factor [30]. The results are shown in Table 4.

It can be seen from the data in Table 4 that the significant level value (*p*) of the model is less than 0.0001, indicating that the model is extremely highly significant and can be used to predict the response value. The correlation coefficient of the model is R2 = 0.9839, which indicates that 98.39% of the lignin yield can be explained by this model. Moreover, the misfit error P is 0.5108, which is not significant, indicating that the model fits the actual data better. The regression coefficients of X_1_, X_2_, X_3_, and X_4_ in the table are extremely significant, indicating that the temperature, time, solid–liquid ratio, and formic acid content have a significant effect on the yield of lignin; the regression coefficients of X_1_X_2_ and X_1_X_3_ are extremely significant, indicating that the interaction term of the reaction temperature and reaction time, as well as the interaction term of the reaction temperature and solid–liquid ratio, both have a significant effect on the yield of lignin; the size of the F value in the table is analyzed as F_X1_ > F_X3_ > F_X2_ > F_X4_, which illustrates that the order of influence on the lignin extract yields is the reaction temperature, solid–liquid ratio, reaction time, and the ratio of formic acid/acetic acid [31].

From the response surface analysis chart, we can visually see the effect of the interaction of influencing factors on the response value Y. 

**Figure 2:**
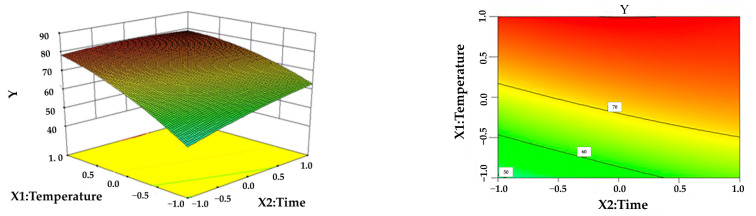
Effect of the interaction between reaction temperature and reaction time on LEY.

Figure 2 shows the effect of the interaction of the reaction temperature and reaction time on the yield of lignin at a solid–liquid ratio of 1:20 and a formic acid content of 80 wt.%. It can be seen from the surface map of Figure 2 that the lignin yield increases rapidly with increasing temperature under a certain reaction time, while the temperature of 60~100 °C increases the yield of lignin slowly, which is also demonstrated by the contour plot at the bottom of the response surface in Figure 2. The lignin yield change is larger in the temperature direction than in the time direction. Therefore, the hydrolysis process should be as short as possible, and the reaction temperature should be appropriately increased to obtain a higher yield [32].

**Figure 3:**
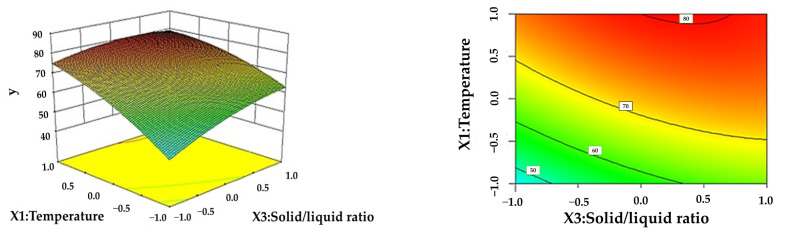
Effect of the interaction between reaction temperature and solid–liquid ratio on LEY.

Figure 3 shows the effect of the interaction of the temperature and solid–liquid ratio on the LEY at a reaction time of 3 h and a formic acid content of 80 wt.%. It can be seen from the surface map of Figure 3 that the lignin yield increases with the increasing temperature or solid–liquid ratio, but the LEY increases rapidly with the temperature direction. The contour line at the bottom of Figure 3 also shows that the gradient of the yield of lignin in the temperature direction is larger than that in the time direction. Therefore, the hydrolysis process should be as appropriate as possible at a lower solid–liquid ratio to obtain a higher yield [33]. 

**Figure 4:**
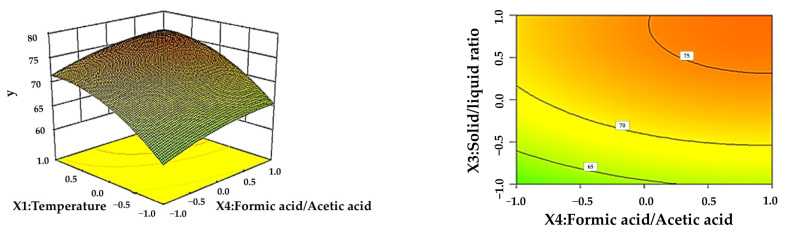
Effect of the interaction between solid–liquid ratio and formic acid content on LEY.

Figure 4 shows the effect of the interaction between the solid–liquid ratio and formic acid content on the LEY at a reaction time of 3 h and a reaction temperature of 80 °C. It can be seen from the surface map of Figure 4 that the yield of lignin rose significantly with increasing temperature under a certain reaction time; the yield of lignin increases with the content of formic acid under certain conditions of the solid–liquid ratio. The contour plot at the bottom of Figure 4 also demonstrates that the LEY is larger in the solid–liquid ratio direction than in the formic acid content, indicating that the solid–liquid ratio has a greater effect on the response than the formic acid [34].

When the LEY was the optimization target, the reaction time, reaction temperature, solid–liquid ratio, and formic acid content were optimized. The design of the above model was optimized by Design-Expert 8.0.5 software. It was found that the reaction time was 3.5 h, the reaction temperature was 90 °C, the solid–liquid ratio was 1:16.5, and the formic acid content was 86.2 wt.%. Under this condition, the highest yield of lignin was 81.24%. To verify the accuracy of this model, the actual experimental verification was carried out under the optimal process conditions predicted by the model [35]. The experimental yield was 79.83%, which was very close to the predicted value of the model, proving that the prediction of lignin yield is very accurate and feasible.

### 3.4. Lignin Extraction Kinetics

The extraction process of lignin from straw is a complex multiphase mass transfer and heat transfer process. When the formic acid/acetic acid solvent is added to the raw material, H^+^ in the solvent penetrates the straw fibers structure by diffusion, and H^+^ destroys the covalent bond between the lignin and the carbohydrates such as cellulose and hemicellulose. The extracted lignin was dissolved in the organic acid solvent, resulting in a poor concentration of lignin inside and outside the straw fibers. Therefore, the concentrated lignin in the straw fibers continuously spread out, and the organic acid solvent continued to enter the interior of the straw fibers [36]. Thus, after repeated mass transfer processes, the concentration of lignin inside and outside the straw fibers was balanced, and the liquid–solid mass transfer balance was achieved.

The process of delignification can be divided into three stages, namely the initial extraction stage, the large delignification stage, and the residual stage. In the initial stage of extraction, the phenolic α-O-4 bond and the phenolic β-O-4 bonds in the lignin are broken; in the large delignification stage, the non-phenolic β-O-4 bonds break mainly; in the residual stage of lignin extraction, the carbon–carbon bond in the molecular structure of lignin is broken, and degradation and condensation reactions occur simultaneously [37,38,39]. Therefore, the lignin extraction process can be described by the following formula:

(6) $$W_{re}=a_{1}\exp(-k_{1}t)+a_{2}\exp(-k_{2}t)+a_{3}\exp(-k_{3}t)$$

In Formula (6), W_re_ is the mass fraction of residual lignin in straw (g/g); a_1_, a_2_, and a_3_ are the initial extraction, large delignification, and residual stage of mass fractions of lignin, and the constants a_1_, a_2_, and a_3_ satisfy: a_1_ + a_2_ + a_3_ = l; k_1_, k_2_, and k_3_ are the rate constants for the delignification reaction of lignin in three processes, respectively.

**Figure 5:**
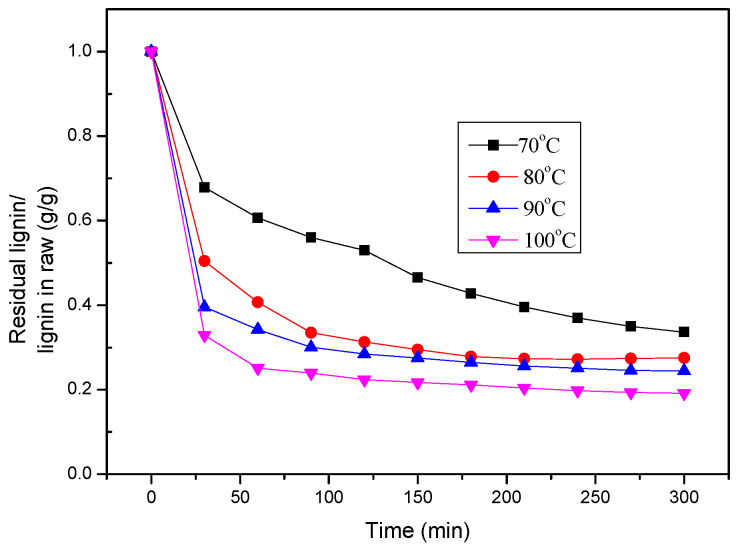
Residual rate of lignin in straw under different temperatures.

It can be seen from Figure 5 that the extraction rate of lignin increased with an increase in reaction temperature, and the change trend of lignin extraction rate at different temperatures was consistent. The extraction of lignin during initial 0–0.5 h was faster, which was a rapid reaction stage; after 0.5 h, the lignin extraction rate changed slowly, which was the residual lignin removal stage.

Because wheat straw was treated in the organic acid system at higher temperatures, the initial extraction process of lignin took a relatively short time, which was much shorter than the manual sampling time [40]. Therefore, the k_1_ in the Formula (6) test approaches −∞. The lignin detachment kinetics Formula (6) can be changed to:

(7) $$W_{re}=a_{1}\exp(-\infty t)+a_{2}\exp(-k_{2}t)+a_{3}\exp(-k_{3}t)$$

In order to study the extraction process of lignin at 70 °C, 80 °C, 90 °C, and 100 °C, the change in residual lignin content in the straw fiber was obtained, as shown in Figure 5.

The kinetic parameters include reaction rate constant, reaction order, adsorption equilibrium constant, etc. The kinetic parameters can be solved by the integral method using experimental data. According to the experimental data obtained under different temperature conditions, regression curve fitting was performed to obtain the following curves [41].

**Figure 6:**
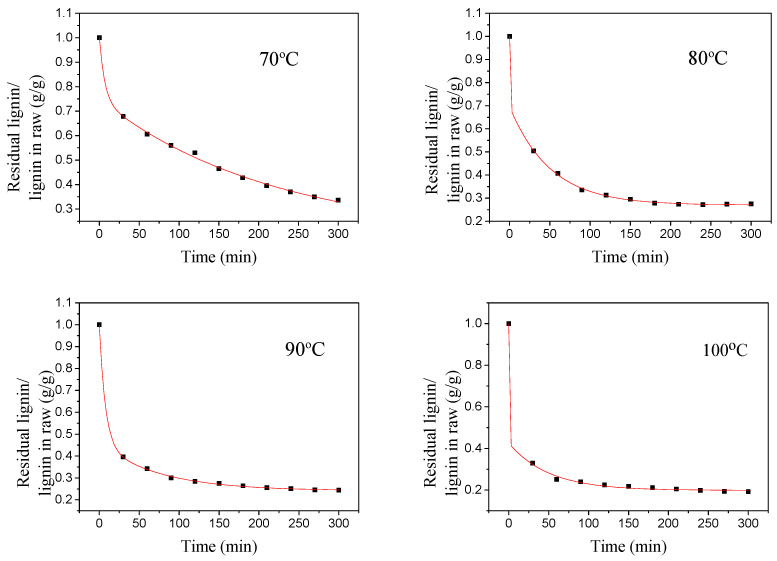
Kinetics curve of delignification at different temperatures.

Figure 6 is a kinetic fit curve of the straw delignification process at different temperatures, as can be seen from Figure 6, and Formula (7) can better describe the entire lignin removal process. The results of the delignification kinetics fitting at different temperatures are shown in Table 5.

It can be seen from the data in Table 5 that the value of k_2_ is much larger than the value of k_3_, indicating that the extraction of lignin mainly occurs in the second stage. As the temperature increases, the values of k_2_ and k_3_ also increase, indicating that the extraction rate of lignin is greatly affected by temperature [42].

### 3.5. Structural Analysis of Extracted Lignin Fractions

**Figure 7:**
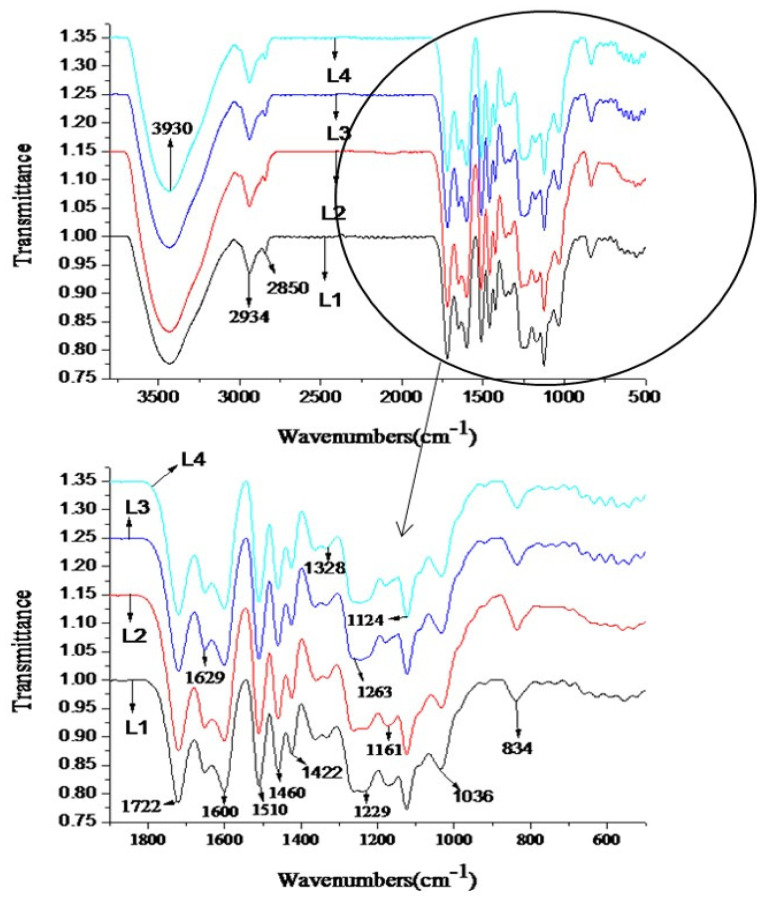
Infrared spectra of the isolated lignin fractions (L1: 80 °C, 3 h, 1:20, formic acid 80%; L2: 90 °C, 3 h, 1:20, formic acid 80%; L3: 80 °C, 4 h, 1: 20, formic acid 80%; L4: 80 °C, 4 h, 1:20, formic acid 90%).

The infrared spectrum can reflect the relative vibration and rotation information between the atoms inside the molecule and then infer the basic structure and characteristics of the material. As can be seen from Figure 7, the strong absorption peak at 3430 cm^−1^ is the O–H stretching vibration of the phenolic hydroxyl group and the aliphatic hydroxyl group, indicating that the extracted lignin polymers contain a large amount of hydroxyl groups. The absorption peaks at 2934 and 2850 cm^−1^ are C–H stretching vibrations of methylene and methyl groups. The absorptions at 1722 cm^−1^ and 1629 cm^−1^ are stretching vibrations of conjugated carbonyl groups; the ones at 1600 cm^−1^ and 1510 cm^−1^ are vibrational peaks of aromatic ring skeleton; the peak at 1460 cm^−1^ is the bending vibration of methylene; the peak at 1422 cm^−1^ is assigned to the vibration of the carbon skeleton of the aromatic ring. The absorption peaks at 1328 cm^−1^ and 1124 cm^−1^ are CO stretching vibrations on the syringyl units; the absorption peak at 1263 cm^−1^ is the carbonyl stretching vibration on the guaiac units; the absorption peak at 1229 cm^−1^ is related to the benzene ring. The weak absorption band at 1161 cm^−1^ may be due to the absorption of ester groups (conjugated), there may be absorption peaks caused by the stretching vibration of p-hydroxyphenyl CO. The absorption peak at 1036 cm^−1^ is caused by the bending vibration in the plane of the aromatic ring CH, and 834 cm^−1^ is the absorption peak caused by the out-of-plane bending vibration of the aromatic ring CH [43].

**Figure 8:**
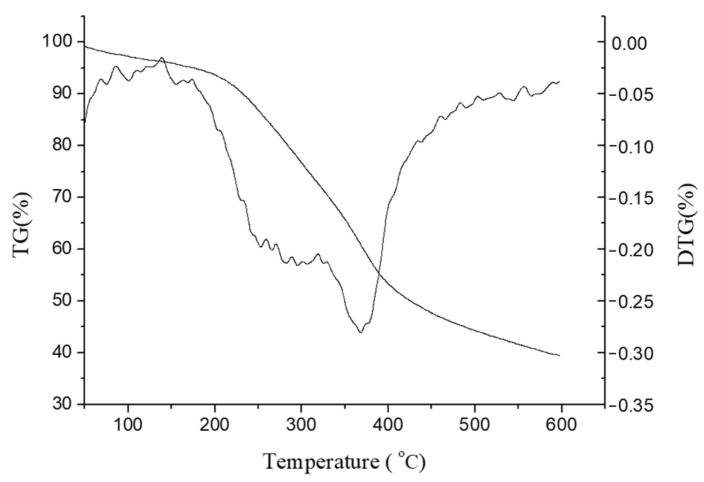
TGA diagram of the extracted wheat straw lignin.

Figure 8 is a TGA graph showing the thermal weight loss curve of wheat straw lignin. It can be seen from the figure that the pyrolysis process of wheat straw lignin is a relatively complicated process, and its pyrolysis range is relatively wide. The weight loss of the lignin can be divided into four stages: the first stage occurs in the range of 40~110 °C, and the thermal weight loss is mainly the evaporation process of adsorbing water. The second stage occurs in the range of 110~200 °C, where the weight loss of lignin is not obvious, and the lignin undergoes glass transition. The third stage occurs in the range of 200~500 °C. At this stage, the weight loss of lignin is more obvious. At 200~400 °C, the lignin component is mainly degraded into volatile gases (CO, CO_2_, CH_4_, etc.), and the lignin is mainly cracked into phenolic substances at 400~500 °C. At the same time, the aldolic acid is rapidly degraded into a gaseous product, and the weight loss rate of lignin reaches the maximum at 365 °C. The fourth stage is at 500~600 °C, the stage of decomposition and re-agglomeration of the aromatic ring. About 40% of the solid residue at about 600 °C is due to the formation of a crosslinked structure in some of the functional groups in the lignin so that the solid residue has high thermal stability [44].

**Figure 9:**
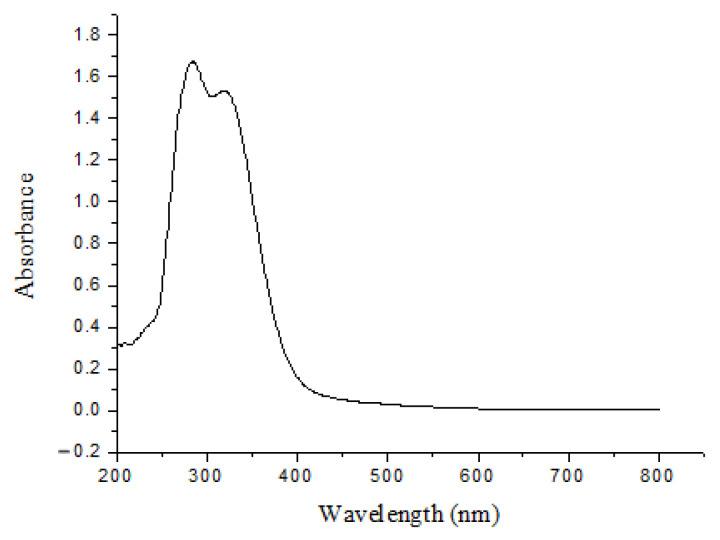
Ultraviolet spectrum of the extracted lignin.

Figure 9 shows the ultraviolet spectrum of the extracted lignin sample. It can be seen from the figure that the maximum absorption peak is at 284 nm, and a shoulder peak appears at 318 nm. At 284 nm, lignin shows the absorption peak caused by π-direction π* electron transition in the aromatic ring. At 318 nm, the electronic transition on the conjugated carbonyl group of lignin aromatic ring is the corresponding absorption peak of ferulic acid and p-coumaric acid. It is indicated that the lignin extracted in this study contains more chemically active groups and has better chemical activity [45].

Overall, a structural analysis of extracted lignin fractions by using FT-IR, TGA, and UV-Vis has proved that the formic acid/acetic acid system did not destroy the chemical structure of the lignin, and the extracted lignin can be used in many fields, as discussed in the introduction.

## 4. Conclusions

The efficient extraction of wheat straw lignin was studied by using the formic acid/acetic acid solvent system, and the optimum experimental conditions for lignin extraction were found: a reaction temperature of 80 °C, a time of 3 h, a solid–liquid ratio of 1:20, and a formic acid content of 80%, and the yield of the obtained lignin was 72.23%. On the basis of a single-factor experiment, the response surface method was used to design and optimize the experimental results. It was found that the temperature had the most significant effect on the yield of lignin in the experimental range, followed by the solid–liquid ratio, reaction time, and formic acid content. During the study of the factor interactions, the interaction between the reaction temperature and solid–liquid ratio had significant effects. After the analysis using the response surface method, the optimum process conditions were a reaction time of 3.5 h, a reaction temperature of 90 °C, a solid–liquid ratio of 1:16.5, and a formic acid content of 86.2%, and the lignin yield under this condition was 79.83%. Some previous studies reported lignin yields above 80–90% with H_2_SO_4_ as the catalyst or hydrogen peroxide post-treatment [46,47]. However, the single treatment using formic acid/acetic acid system in this study has some advantages; for example, formic acid and acetic acid are cheap solvents and can be reused, and the single extraction process is mild under normal pressure and temperature conditions.

## Figures and Tables

**Table 1:** Response surface factor level of lignin extraction.

Level	X_1_ Temperature (°C)	X_2_ Time (h)	X_3_ Solid–Liquid Ratio (g/mL)	X_4_ Formic Acid Content (%)
−2	60	1	1:10	60
−1	70	2	1:15	70
0	80	3	1:20	80
1	90	4	1:25	90
2	100	5	1:30	100

**Table 2:** CCD design experimental scheme and response value.

Assay	X_1_	X_2_	X_3_	X_4_	Y/%
1	0	2	0	0	75.18
2	−2	0	0	0	35.77
3	1	1	−1	−1	69.56
4	0	0	−2	0	47.91
5	0	0	0	0	72.71
6	0	−2	0	0	60.21
7	0	0	0	0	70.45
8	1	−1	1	−1	72.46
9	0	0	0	−2	63.22
10	1	1	1	−1	75.61
11	0	0	0	0	73.45
12	−1	1	−1	1	52.37
13	−1	1	1	−1	64.54
14	1	−1	1	1	76.86
15	−1	−1	−1	−1	34.47
16	1	−1	−1	−1	73.56
17	−1	−1	1	−1	48.92
18	0	0	0	2	72.56
19	0	0	2	0	74.06
20	−1	−1	1	1	56.12
21	1	−1	−1	1	75.06
22	−1	1	−1	−1	48.83
23	−1	−1	−1	1	39.78
24	1	1	−1	1	74.37
25	1	1	1	1	79.01
26	2	0	0	0	80.64
27	−1	1	1	1	68.82

**Table 3:** Chemical composition of wheat straw.

Cellulose (%)	Hemicellulose (%)	Lignin (%)	Ash (%)
48.34	22.64	18.86	2.56

**Table 4:** Response surface model analysis results.

Source	Sum of Square	Degree of Freedom	Mean Square	F Value	Significant Level (*p*)
model	4902.32	14	350.17	114.60	<0.0001
X_1_	3091.29	1	3091.29	1011.69	<0.0001
X_2_	306.88	1	306.88	100.43	<0.0001
X_3_	668.24	1	668.24	218.69	<0.0001
X_4_	118.37	1	118.37	37.84	<0.0001
X_1_X_2_	186.73	1	186.73	61.11	<0.0001
X_1_X_3_	166.15	1	166.15	54.38	<0.0001
X_1_X_4_	2.42	1	2.42	0.79	0.3912
X_2_X_3_	8.07	1	8.07	2.64	0.1302
X_2_X_4_	0.35	1	0.35	0.12	0.7395
X_3_X_4_	1.06	1	1.06	0.35	0.5666
X_1_^2^	275.36	1	275.36	90.12	<0.0001
X_2_^2^	31.76	1	31.76	10.40	0.0073
X_3_^2^	179.13	1	179.13	58.62	<0.0001
X_4_^2^	28.72	1	28.72	9.40	0.0098
Residual	36.67	12	3.06	—	—
Misfit error	31.78	10	3.18	1.30	0.5108
Pure error	4.89	2	2.44	—	—
sum	4938.99	26	—	—	—

Note: *p* < 0.001 indicates the difference is extremely significant; *p* < 0.01 indicates the difference is highly significant; *p* < 0.05 indicates the difference is significant.

**Table 5:** Kinetics fitting results of delignification at different temperatures.

Temperature Reflex/°C	k_1_/min^−1^	k_2_/min^−1^	k_3_/min^−1^	R^2^
70	—	0.1536	0.0052	0.9874
80	—	0.2840	0.0097	0.9943
90	—	0.5342	0.0163	0.9974
100	—	1.2391	0.0275	0.9945

## Data Availability

The original contributions presented in the study are included in the article, further inquiries can be directed to the corresponding authors.
